# Object-Oriented Hierarchy Radiation Consistency for Different Temporal and Different Sensor Images

**DOI:** 10.3390/s18030682

**Published:** 2018-02-25

**Authors:** Nan Su, Yiming Yan, Chunhui Zhao, Liguo Wang

**Affiliations:** Department of information engineering, Harbin Engineering University, Harbin 150001, China; zhaochunhui@hrbeu.edu.cn (C.Z.); wangliguo@hrbeu.edu.cn (L.W.)

**Keywords:** different temporal and different sensor images, illumination consistency, smoothness consistency, dense matching in 3D reconstruction

## Abstract

In the paper, we propose a novel object-oriented hierarchy radiation consistency method for dense matching of different temporal and different sensor data in the 3D reconstruction. For different temporal images, our illumination consistency method is proposed to solve both the illumination uniformity for a single image and the relative illumination normalization for image pairs. Especially in the relative illumination normalization step, singular value equalization and linear relationship of the invariant pixels is combined used for the initial global illumination normalization and the object-oriented refined illumination normalization in detail, respectively. For different sensor images, we propose the union group sparse method, which is based on improving the original group sparse model. The different sensor images are set to a similar smoothness level by the same threshold of singular value from the union group matrix. Our method comprehensively considered the influence factors on the dense matching of the different temporal and different sensor stereoscopic image pairs to simultaneously improve the illumination consistency and the smoothness consistency. The radiation consistency experimental results verify the effectiveness and superiority of the proposed method by comparing two other methods. Moreover, in the dense matching experiment of the mixed stereoscopic image pairs, our method has more advantages for objects in the urban area.

## 1. Introduction

With the development of satellite imaging for Earth observation, it is feasible to obtain high-resolution multi-angle, multi-temporal, and multi-sensor satellite data. A highly precise 3D reconstruction by making full use of the multi-source data is one of the important research hotspots in the remote sensing field [1]. Especially in urban areas, 3D information of building objects is recovered accurately, which is very helpful for people’s lives, and includes 3D positioning, 3D navigation, land, and resources monitoring. In the 3D reconstruction field, the traditional stereoscopic image pairs are obtained by the same sensor at almost the same time. Unfortunately, the mixed stereoscopic image pairs of different temporal and different sensors have different radiation characteristics, which strongly affect dense matching precision in the 3D reconstruction. Different temporal images are obtained at different illumination conditions, resulting in the difference in the grayscale level between the stereo image pairs. Different sensor images have different noise levels. Moreover, the different illumination and different noise levels are important factors in the performance of the dense matching method based on area. However, to produce highly accurate 3D reconstruction of surfaces, there is a significant lack of radiation consistency method for the mixed stereoscopic image pairs of different time and different sensor. In the paper, we comprehensively analyze the influence factors for the dense matching of different time and different sensor data, and firstly propose a novel hierarchy radiation consistency method to simultaneously obtain the illumination consistency and the smoothness consistency of the mixed stereoscopic image pairs.

For the mixed stereoscopic image pairs of different temporal, there is no specific radiation consistency method to solve the problem of different illumination for dense matching. However, several methods have been developed for the radiation consistency of multi-temporal remote sensing images in other applications, such as change detection and image mosaicking [2,3]. In general, there are two types of radiation consistency method, namely, absolute normalization and relative normalization [4]. The absolute radiometric normalization mainly depends on the parameters of the environment and sensor. Therefore, the paper focuses more on the relative normalization method to obtain a similar grayscale characteristic between the different temporal images. Zhang proposed to improve contrast and brightness for the illumination normalization method, which is a multilevel processing method for different illuminations [3]. Most relative radiometric normalization methods are based on the assumption that the invariant pixels in the same area of the different temporal images are spatially homogeneous and approximated by a linear relationship [5]. Canty et al. [6] proposed the multivariate alteration detection method (MAD) to obtain the invariant pixels for building the linear relationship. Nielsen [7,8] further proposed the iteratively reweighted MAD method (IRMAD) to demonstrate a great performance for relative radiometric normalization. Zhang et al. [9] used iterative slow feature analysis (ISFA) extract invariant features for radiation consistency. Zhong [10] proposed hierarchical regression to build the more accurate linear relationship for suppressing the negative effect of change pixels. For the application of change detection and image mosaicking, all the above relative normalization methods only adjust the global grayscale consistency between different temporal images. For the dense matching of 3D reconstruction, the grayscale consistency of details in object areas is more important. Besides, the mentioned methods only notice the relative consistency between different temporal images to ignore the radiation quality of each image, which has a significant impact on dense matching. 

For the traditional stereoscopic image pairs of the same sensor, several literatures pointed out that noise has an important influence on dense matching. Crespi et al. used CARTOSAT-1 satellite stereoscopic image pairs to analyze the influence of noise distribution on dense matching in 3D reconstruction and proposed that image denoising is helpful [11,12]. Pateraki and Poli [13,14] both introduced imaging principle of different TDI-CCD sensor stereoscopic image pairs leading to different noise levels and also used the Wallis filter method for image denoising to improve dense matching. The mentioned methods above all demonstrated image denoising being beneficial for dense matching in 3D reconstruction. For the mixed stereoscopic image pairs of different sensors, Eisenbeiss [15] indicated that different smoothness levels between different sensor images result in the low accuracy of dense matching in DSM generation. In the recent research, Aguilar [16] analyzed the causes that affect the dense matching in detail by multiple sets of mixed stereoscopic image pairs. The paper [16] also arrived at a conclusion that different illumination and different smoothness seriously affected the accuracy of dense matching. The above paper both analyzed the influence factors that different noise levels were leading to the low accuracy of dense matching. However, no one gave a method for the mixed stereoscopic image pairs of different sensors to simultaneously control two image noise levels, which can obtain the smoothness consistency images. Therefore, the purpose of smoothness consistency in our paper is to achieve a similar noise level for different sensor stereo image pairs.

The purpose of our paper is to improve dense matching for different time and different sensor stereoscopic image pairs. Moreover, dense matching is a crucial step in 3D reconstruction. In other words, better results of dense matching can result in better 3D reconstruction. In this paper, there are three main contributions. Firstly, we have comprehensively analyzed various influencing factors of different temporal and different sensor images for dense matching. Additionally, the hierarchy radiation consistency method is proposed to solve illumination consistency for different temporal images and smoothness consistency for different sensor images. Secondly, the object-oriented algorithm idea is proposed to obtain the radiation consistency in more detail, which is especially helpful for dense matching of urban areas. Finally, the smoothness consistency method using union group sparse is first proposed to obtain similar smoothness levels between different sensor stereoscopic image pairs. Therefore, our hierarchy radiation consistency method is beneficial for 3D reconstruction.

The remainder of this paper is organized as follows. Section 2 shows the whole flow of the proposed method. In Section 3, object region association method is described. We introduce the proposed illumination consistency method and smoothness method in detail in Section 4 and Section 5, respectively. Our experiment results are showed by comparing two other methods in Section 6, and the conclusion in Section 7.

## 2. Proposed Methods 

**Figure 1:**
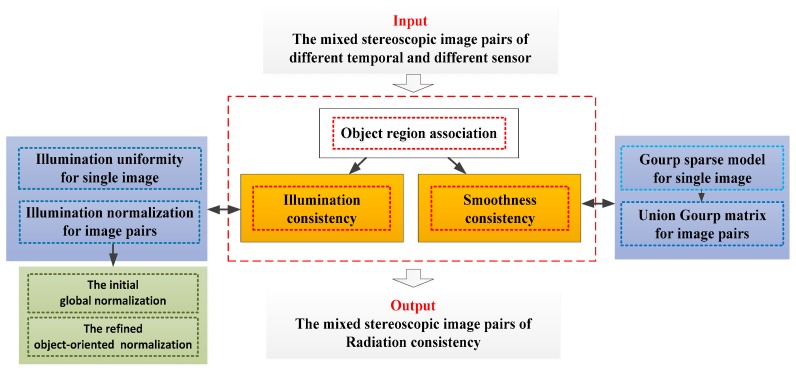
The workflow of the proposed method.

The purpose of the proposed object-oriented hierarchy radiation consistency method is to obtain radiation consistency of different time and different sensor images for improving area-based dense matching. As shown in Figure 1, each level of our method is marked by different colors. In the paper, radiation consistency is the top level concept. We think that the influence factors of radiation consistency are mainly composed of two points, which are the illumination consistency and the smoothness consistency. Different temporal images are acquired by different illumination conditions to cause images’ different brightnesses, while different sensor images are obtained by different design TDI-CCD sensors to cause images’ different smoothnesses. This is the first level in the yellow boxes of Figure 1.

For smoothness consistency, we propose union group matrix to simultaneously control the smoothness levels to be similar for the mixed stereoscopic image pairs. For illumination consistency, we propose a two-layer method to solve illumination uniformity for a single image and illumination normalization for image pairs, respectively. In the illumination uniformity step, the relative gradient method is employed to improve the radiation quality of single image. This is the second level in the blue boxes of Figure 1.

For illumination normalization, coarse-to-fine relative normalization method is proposed. In the coarse step, the improved singular value equalization is employed for the initial global normalization. In the fine step, an object-oriented illumination normalization method based on piecewise linear correction is proposed to adjust the details for each object. This is the third level in green boxes of Figure 1.

In addition, object region association is the premise of the whole object-oriented method. For object region association, the remote sensing image segmentation method based on occlusion random texture model (ORT) is employed to extract the object areas of each image [17]. Then, sparse matching method based on SIFT feature is used to build the relationship between the same object areas of the mixed stereoscopic image pairs [18]. It can recover the radiation consistency of different temporal and different sensor images in more detail. In the following sections, the detail of the algorithm is provided.

## 3. Object Region Association

For high-precision 3D reconstruction, the accuracy of dense matching in object areas is extremely critical factor. In the urban areas, it is difficult to obtain successful matching due to the uncontinuous elevation information of building objects. Moreover, the mixed stereoscopic image pairs of different time and different sensors have different radiation properties, which are almost impossible to dense match, especially in the object areas. Therefore, the paper proposed a novel grayscale consistency method, which not only considered the whole image but also mainly focused on object-oriented uniformity. It is very helpful for the accuracy of dense matching in particular with object areas.

Object extraction is a significant part in our object-oriented method. McCann et al. [17] proposed the segmentation algorithm based on occlusion random texture model, which has an excellent performance in the unsupervised segmentation. Moreover, there are corresponding improved methods for extracting objects in remote sensing image processing. Yuan [19] proposed to use the local histogram and linear regression. Shu [20] presented non-negative, low-rank sparse correlation mapping to use in the remote sensing image segmentation. In the paper, the method proposed by [20] is employed, which obtained high precision for the build object segmentation results. Based on object segmentation results, feature matching is used to build relationships between corresponding object areas in the mixed stereoscopic image pairs. The SIFT (Scale Invariant Feature Transform) feature matching method [18] can be overcome by the influence of some illumination and noise to achieve accurate sparse matching results robustly. Therefore, when the number of matching points by SIFT are more than the threshold (threshold is 2 in the paper) in every extracted object area, the two object areas in the stereo image pairs are considered to be matched in the paper. If no matching points exist in extracted object areas, the entire region of the extracted building object in the reference image is treated as a template matching window to be associated. In a word, the extracted object areas in the stereo image pairs are all established the corresponding relationship.

## 4. Illumination Consistency for Different Temporal Images

The multi-temporal data is obtained by different illumination conditions and is the most characteristic. Moreover, different illuminations must lead to the difference in grayscale characteristics between the multi-temporal stereoscopic image pairs. It will increase the difficulty of stereo matching, which will have a significant influence on the accuracy of 3D reconstruction, especially in the object areas. In the paper, we proposed to adjust the illumination difference in two ways. On the one hand, the relative gradient method is employed to obtain illumination uniformity for a single image. On the other hand, illumination normalization method from global to objects is proposed to eliminate the grayscale difference between the multi-temporal mixed stereoscopic image pairs.

### 4.1. Illumination Uniformity for Single Image

The purpose of this paper is to improve the radiation quality of remote sensing image for dense matching and 3D reconstruction. In fact, different illumination is only one of the factors influencing different radiation characteristics of multi-temporal images. The overexposure or overshadow of the image local areas also has a great impact on the matching. Therefore, the relative gradient is used to improve the contrast and texture details of the object areas for illumination uniformity images, which are very helpful for dense matching. The original gradient of the image is adjusted to make the larger gradient suppressed and the smaller gradient stretched. The adjusted gradient can be expressed as

(1) G(x,y)=∇I(x,y)Θ(x,y)

in which ∇I(x,y) represents the original gradient of the image. G(x,y) is the improved gradient. Θ(x,y) is an adjustment function, and the value depends on the local relative gradient. The relative gradient is shown as

(2) $$\left\|\nabla I^{\prime }(x,y)\right\|=\frac{\left\|\nabla I(x,y)\right\|}{f(I(x,y)*g(x,y))}$$

in which g(x,y) is gaussian kernel, and * represents convolution operation. The convolution operation using the original image and gaussian kernel is to inhibit edge gradient mutation, which results in enhanced image edges to be unnatural, especially in the object edge areas. Therefore, the adjustment function Θ(x,y) is computed by the relative gradient based on the following formulation.

(3) $$\Theta (x,y)={\left(\frac{\mu \cdot \bar{\left\|\nabla I^{\prime }(x,y)\right\|}}{\left\|\nabla I^{\prime }(x,y)\right\|}\right)}^{1-\xi }$$

in which *µ* is a constant, it is set to 0.1, and $\bar{\left\|\nabla I^{\prime }(x,y)\right\|}$ is the average of the relative gradient. In other words, $\mu \bar{\left\|\nabla I^{\prime }(x,y)\right\|}$ is the gradient threshold. The gradient being less than the threshold is stretched, and the gradient being larger than the threshold is suppressed. For example, the overexposure object areas apply to smaller value, while the overshadow areas apply to larger value. ξ controls the amplitude of gradient adjustment (it is set 0.8 in the paper). The illumination uniformity images can be reconstructed by the improved gradient G(x,y).

### 4.2. Illumination Normalization for Stereoscopic Image Pairs

Illumination normalization to eliminate the difference of grayscale characteristics between the multi-temporal stereoscopic image pairs is an important part in the paper. This part is equal to relative radiation normalization in many types of research, which only focus on relative grayscale consistency of multi-temporal data. In early studies, many researchers used histogram matching and gamma collection method to obtain similar gray statistics of images. In recent years, for change detection and images mosaic, many people proposed that objects in the same area of different temporal images constitute spatial homogeneity, which is assumed to be the linear relationship. Based on these studies, this paper proposed two stages of illumination normalization algorithm from global to objects. Firstly, the global illumination consistency is corrected based on grayscale statistics information. Secondly, the gray values of object areas are further refined by the linear transformation relationship using object region association results.

#### 4.2.1. The Initial Global Illumination Normalization 

The improved singular value equalization is employed to obtain the global illumination normalization results. The singular value equalization is used based on *SVD* (Sigular Value Decomposition, *SVD*) [21]. For any image I, its singular value decomposition can be expressed as

(4) $$I=D_{I}\Sigma_{I}V_{I}^{T}$$

in which *D*, *V*, and Σ are SVD decomposition matrix of *I*. *D* and *V* are both Orthogonal Matrix. The column of *D* and *V* are left-singular vectors and right-singular vectors of *I*, respectively. $\Sigma_{I}$ is the diagonal matrix including the singular value order, which is the main information of the images. Additionally, the larger value of the singular value in the main diagonal is in the front, which is the greater contribution to the image. It is the basic of image reconstruction by SVD. The original singular value equalization method make the grayscale mean of different temporal images closer to max/2, which can obtain grayscale consistency and enhancement. However, the illumination uniformity method has been used to produce better enhanced images. The max/2 is replaced with the grayscale mean of multi temporal images by the illumination uniformity method in the paper. Assuming that *A* is the grayscale mean of multi-temporal mixed stereoscopic images. The new image $I_{A}$ with single grayscale value *A* is singular value decomposed. Using the max singular value $\max(\Sigma_{I_{A}})$, the image transform coefficient of global illumination normalization λ is computed as

(5) $$\lambda =\frac{\max(\Sigma_{I_{A}})}{\max(\Sigma_{I})}$$

in which the mean of $I_{i}$ is less than *A*, and λ is larger than 1. Otherwise, λ is smaller than 1. Moreover, the new singular value matrix is constructed by using this transformation coefficient $\hat{\Sigma_{I}}=\lambda \Sigma_{I}$. The improved image $\hat{I}$ by global illumination normalization can be reconstructed by the new singular value matrix, as shown in (6)

(6) $$\hat{I}=D_{I}\hat{\Sigma_{I}}V_{I}^{T}$$

#### 4.2.2. The Refined Object-Oriented Illumination Normalization

In the field of study on the relative radiation-normalization of different temporal data, many researchers like Zhang [9] and Zhong [10] have proposed the assumption that the invariant pixels are linear relationship under different illumination conditions. Under the assumption, they focus on the study of the invariant pixels extraction. The more accurate linear relationship is established by extracting a large number of invariant pixels and removing the varying pixels in the changed areas. In the paper, the corresponding object areas have been associated. The feature matching points in the matching object areas are just the invariant pixels. Therefore, we proposed the object-oriented refined illumination normalization to establish a piecewise linear relationship for every object, which only needs few invariant pixels in the same object and avoid changing pixels. Based on the matching points of a single object, the linear relationship can be expressed as

(7) $$y_{k}^{m}=\lambda_{m}x_{k}^{m}+\xi_{m}$$

in which *m* represents the number of object areas, *k* is the position of the pixel, *y* is the gray value of the reference image, and *x* is the gray value of different temporal image in the corresponding position. λ and ξ represent linear coefficients, which are the purpose of solving linear function. The predicted gray value of the transformed image is shown as (8)

(8) $${\hat{y}}_{k}^{m}={\hat{\lambda }}_{m}x_{k}^{m}+\hat{\xi }$$

in which ${\hat{\lambda }}_{m}$ and ${\hat{\xi }}_{m}$ are the linear coefficients predicted value, which are computed by the matching points in the same object $x_{k}^{m}$ and $y_{k}^{m}$.

Because each matching object area has multiple matching feature points to calculate the linear coefficients, ${\hat{\lambda }}_{m}$ and ${\hat{\xi }}_{m}$ are obtained by weight least squares method. For every matching object area, the linear coefficients are computed by minimizing the energy function based on the following formulation.

(9) $$({\hat{\lambda }}_{m},{\hat{\xi }}_{m})=\underset{\lambda ,\xi }{\mathrm{argmin}}\sum_{k}^{N}\tau_{k}\cdot {\left|y_{k}-\lambda x_{k}-\xi \right|}^{2}$$

in which *N* is the number of matching points in every matching object area, and $\tau_{k}$ is the weight of the kth pixel. To the weight $\tau_{k}$, the reference image and the transform image need to be normalized, which can avoid the loss of radiation resolution. It is shown as (10).

(10) $$\begin{array}{c} {x^{\prime }}_{k}=\frac{\left(x_{k}-\mu_{x}\right)}{\sigma_{x}}\\ y_{k}^{\prime }=\frac{\left(y_{k}-\mu_{y}\right)}{\sigma_{y}} \end{array}$$

in which $\mu_{y}$ $\sigma_{y}$ $\mu_{x}$ $\sigma_{x}$ represent the mean and the standard deviation of the reference image and the transform image, respectively. After the normalization, the weight of each pair feature matching points mainly depends on the difference of gray values $\left|{y_{k}}^{\prime }-{x_{k}}^{\prime }\right|$. Let T represent the difference $\left|{y_{k}}^{\prime }-{x_{k}}^{\prime }\right|$, and the weight $\tau_{k}$ can be computed as

(11) $$\tau_{k}=1-\frac{T_{k}-\min\{T_{0},T_{1}\ldots T_{N}\}}{\max\{T_{0},T_{1}\ldots T_{N}\}-\min\{T_{0},T_{1}\ldots T_{N}\}}$$

From the formulation (11), $\tau_{k}$ is between 0 and 1. Moreover, the difference $\left|{y_{k}}^{\prime }-{x_{k}}^{\prime }\right|$ is larger, and the weight $\tau_{k}$ is smaller, although SIFT feature matching owns the high accuracy. To obtain the accurate linear relationship, some mismatched feature points should be avoided to participate in computation of linear coefficients as the invariant pixels. Because the linear relationship of each object is calculated independently, the disparity in the same object has the same value. The highest frequency disparity is considered as the truth. Matching points that are too different from the truth will be removed. Satisfying the formula (12), the matching points are considered to be the invariant pixels.

(12) $$\left|D_{k}^{m}-\bar{D^{m}}\right|<l$$

in which $D_{k}^{m}$ represent the disparity of *k*th pixel in *m*th matching object area, and $\bar{D^{m}}$ represent the highest frequency disparity in *m*th matching object area. The value of the highest frequency disparity ($\bar{D^{m}}$) depends on the height of each building object. The value of $\bar{D^{m}}$ is the most normal value, which is equal to the “truth” for the disparity of each building object. The purpose of $\bar{D^{m}}$ is to remain the correct matching points and to eliminate the error matching points. *l* is the threshold. It is 2 in the paper.

## 5. Smoothness Consistency for Different Sensor Images

Satellite panchromatic images are almost obtained by TDI-CCD. The different satellite sensors used TDI-CCD with different storage units designed by different companies, which can lead to the different smoothness levels of images. Almost any reference shave mentioned that the noise levels affect dense matching results. The purpose of smoothness consistency in the paper is to achieve similar noise level for different sensor stereo image pairs. We propose a method based on simultaneously controlling two image noises on group sparse representation for the different sensor stereoscopic image pairs. 

### 5.1. Group Sparse Model

The sparse model performed excellently in the image restoration, which can suppress noise and protect image structure features. However, in the traditional sparse representation, the patches are independent and cannot build a relationship between similar patches belonging to the same objects [22,23]. Moreover, the sparse model based on patches cannot establish a connection between the different sensor images. In group sparse method, the basic unit of sparse representation for the single image is the group. Each patch extracted is matched a set of nonlocal patches with similar structure. All the similar patches are stacked to construct a group. This group matrix contains all the similar structure patches to establish their connection spatial adaptively [24]. 

**Figure 2:**
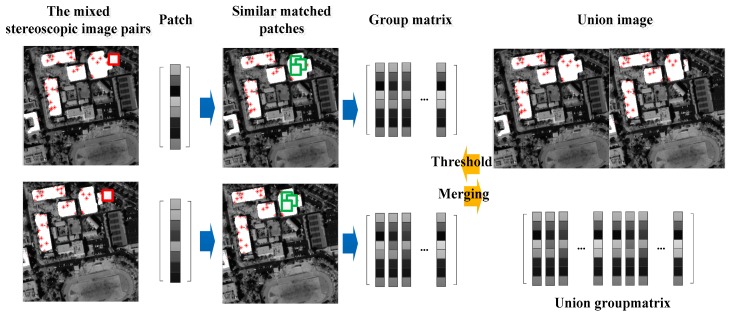
The proposed smoothness consistency method based on union group sparse in the paper.

For each patch $i_{ks}$, with size $\sqrt{p_{s}}*\sqrt{p_{s}}$, we search its *t* best matched patches in the nonlocal space of the single image. $i_{G_{k}}$ is named as a group with size $p_{s}*t$ containing all the matched patches with similar structures. Figure 2 shows that each patch $i_{ks}$ is represented as a vector, and the group $i_{G_{k}}$ is integrated to a matrix. Analogous to the sparse coding in the patch space, the group $i_{G_{k}}$ is expressed as $i_{G_{k}}=D_{G_{k}}\alpha_{G_{k}}$ by a dictionary $D_{G_{k}}$ and sparse vectors $\alpha_{G_{k}}$. According to this concept of group, the whole image can be expressed by structured sparsely code as (13).

(13) $$I=D_{G}\circ \Lambda_{G}={\left(\sum_{k=1}^{n}R_{G_{k}}^{T}R_{G_{k}}\right)}^{-1}\sum_{k=1}^{n}R_{G_{k}}^{T}(D_{G_{k}}\alpha_{G_{k}})$$

in which $D_{G}$ and $\Lambda_{G}$ denote the concatenation of all $D_{G_{k}}$ and $\alpha_{G_{k}}$, respectively. $R_{G_{k}}$ is actually an operator that extracts the group $i_{G_{k}}$ from the single image *I*, which means $i_{G_{k}}=R_{G_{k}}(I)$. Additionally, its transpose is denoted by $R_{G_{k}}^{T}$. *n* is the number of groups, and it is obvious that each patch corresponds to a group. Like traditional sparse algorithm, group sparse model can be formulated into the following minimization problem [25,26]:

(14) $$\begin{array}{l} (D_{G_{k}},\alpha_{G_{k}})=\underset{\alpha_{G_{k}}}{\arg\min}{\left\|i_{G_{k}}-D_{G_{k}}\alpha_{G_{k}}\right\|}_{2}^{2}+\tau {\left\|\alpha_{G_{k}}\right\|}_{1,2}\\ {\left\|\alpha_{G_{k}}\right\|}_{1,2}=\sum_{i=1}^{K}\sqrt{\alpha_{i,1}^{2}+\alpha_{i,2}^{2}+\ldots +\alpha_{i,m}^{2}} \end{array}$$

in which the group sparsity defined by a pseudo-matrix norm ${\left\|\alpha_{G_{k}}\right\|}_{p,q}$ is considered, and in the paper set *p* = 1, *q* = 2. The group sparsity regularizer ${\left\|\alpha_{G_{k}}\right\|}_{1,2}$ is the sum of standard deviations associated with sparse coefficient vector. $\alpha_{G_{k}}=[\alpha^{1},\alpha^{2},\ldots ,\alpha^{n}]$ is the sparse vector, the sparse coefficients $\alpha^{i}=[\alpha_{i,1},\alpha_{i,2},\ldots ,\alpha_{i,m}]$ are the *i*th row of matrix $\alpha_{G_{k}}$, and *m* = *t* + 1 denote *t* matched patches $i_{ks}$. In this paper, we apply SVD to obtain the self-adaptive learning dictionary for each group to form a standard low-rank approximation problem as [27], rather than a given dictionary *D* for the entire image in the traditional sparse algorithm.

(15) $$(D_{G_{k}},\Sigma_{G_{k}},V_{G_{k}})=\underset{D_{G_{k}}\Sigma V}{\arg\min}{\left\|i_{G_{k}}-D_{G_{k}}\Sigma_{G_{k}}V_{G_{k}}^{T}\right\|}_{2}^{2}+\tau \overset{K}{\underset{k=1}{\Sigma }}\lambda_{i}$$

(16) $$\left\{ \begin{array}{l} \left(D_{G_{k}},\Sigma_{G_{k}},V_{G_{k}})=SVD(i_{G_{k}}\right)\\ {\hat{\Sigma }}_{G_{k}}=S_{\tau }(\Sigma_{G_{k}}) \end{array}\right.$$

in which $\Sigma =diag\{\lambda_{1},\lambda_{2},\ldots ,\lambda_{K}\}\;K=\min\left(m,n\right)$ is a diagonal matrix, and $S_{\tau }$ represents the soft thresholding operation. The predicted denoised image is obtained by $\hat{I_{G}}=D_{G}\hat{\Sigma_{G}}V_{G}$.

### 5.2. Union Group Sparse Method for the Smoothness Consistency of Different Sensor Images

Many researchers refer to the effect of noise on dense matching. Moreover, the mixed stereoscopic image pairs are obtained by different TDI-CCD sensors with different designs (such as different storage units, different sensor calibration) leading to different smoothness levels, which have more of an effect on dense matching. We propose union group matrix to simultaneously suppress the noise of different sensor stereoscopic image pairs for the same smoothness level, which makes use of extracting similar patches in the group sparse model to build the relationship between two different sensor images. The proposed method is as shown in Figure 2. The red * are sparse matching points by SIFT method. The red square is the reference central image patch, and the green squares are the matched image patches. Each patch is denoted by the vector. All the matched patches are stacked in the form of matrix to construct the group. The two group matrices are obtained from the same objects of different sensor images at the same time. The union group matrix is constructed by the two matrices with the similar structure patches in the same objects, because the two different sensor images have similar grayscale information after our illumination consistency method. In other words, the two diagonal matrices have similar values. The sparse model is solved by the SVD, while the union group matrix can make the sparse model use the same threshold union *τ*. The same threshold *τ* and the similar diagonal matrices can make two different sensor reconstructed images keep the similar information, which can obtain the same smoothness level images as much as possible. 

In order to construct a union group matrix, the corresponding group matrices of two different sensor images with similar patches are obtained at the same time. Assuming that, the two different sensor images are s1 and s2. Any patch of the reference image s1 in the matching object areas are considered as central patch. The rules that construct the union matrix mainly include the following two points. (a) The central patches of s2 are obtained on the corresponding position with the distance $\bar{D^{m}}$ apart from the central patch of s1 in the s2 image. The $\bar{D^{m}}$ is the highest frequency disparity in the matching object areas. (b) The similar patches of the central patch are searched only in the matching object areas. Therefore, the union group matrix is obtained by the two central patches and its own similar patches from two different sensor images, which all belongto the same objects that have similar structure information. The size of the patches is 64 and the number of the similar patches is 30 in our paper. 

The group sparse model as standard low-rank approximation problem is solved by SVD, whose core problem is to determine the threshold τ of the singular value matrix. Following the paper [25], for the given noisy image, the threshold τ can be $\tau_{i}=2\sqrt{2}\sigma_{\omega }^{2}/\sigma_{i}$. $\sigma_{i}$ denotes the local estimation changes, and $\sigma_{\omega }$ is a given initial value denoting the global changes of the given image. The update formula of $\sigma_{i}$ and $\sigma_{\omega }$ are shown as (17) and (18), respectively.

(17) $${{\hat{\sigma }}_{i}}^{\left(k+1\right)}=\sqrt{max({\left(\lambda_{i}^{\left(k\right)}\right)}^{2}/m-{\left(\sigma_{\omega }^{\left(k\right)}\right)}^{2},0)}$$

(18) $${\hat{\sigma }}_{\omega }^{\left(k\right)}=\gamma \sqrt{\sigma_{\omega }^{2}-{\left\|Y-Y^{\left(k+1\right)}\right\|}_{l2}}$$

in which $\lambda_{i}$ is the singular value of union group matrix, *m* is the number of columns in the union group matrix, and *Y* is the original union image. $Y^{\left(k+1\right)}$ represents the estimated union denoised image after (*k* + 1) iteration. The $\sigma_{i}$ and $\sigma_{\omega }$ of union group matrix are updated by the union image updated. The same union threshold *τ* is used to threshold the singular value matrix of two original group matrices, which can control the smoothness level of two different sensor images at the same time. The final denoised images are obtained based on iterate regularized, which makes the filtered noise of each iteration feedback to the image. It can be expressed as

(19) $$y^{\left(k+1\right)}={\hat{y}}^{\left(k\right)}+\delta (y-{\hat{y}}^{\left(k\right)})$$

in which *y* is the original image, and $y^{\left(k+1\right)}$ is the *k* + 1 iteration result.

## 6. Experimental Results

### 6.1. Dataset Description

To evaluate the performance of the proposed method, we conducted three groups of experiments on the mixed stereoscopic image pairs of different time and different sensor. The first and second groups of data are both WorldView-2 and QuickBird images. The mixed stereoscopic image pairs are from different time and different sensors. The study scene is different areas over San Francisco in the USA. The WorldView-2 images were acquired on the 9 October 2011 with 0.5 m resolution, and the QuickBird images were the 11 November 2007 with 0.6 m resolution. In our experiments, the images were resampled to 0.5 m resolution. The last data is different temporal BJ-2 satellite images with 1 m resolution over Beijing in China. The images were taken on different months in 2016 from 21st-century space Technology Application Co.,s Ltd. (New Taipei City, Taiwan). All the experimental stereoscopic image pairs have different illumination properties and some have different noise levels. The mixed stereoscopic image pairs are processed to obtain radiation consistency for dense matching, which demonstrates the efficiency and robustness of the proposed algorithm.

### 6.2. Comparative Experiment of Radiation Consistency for Multiple Mixed Stereoscopic Image Pairs

**Figure 3:**
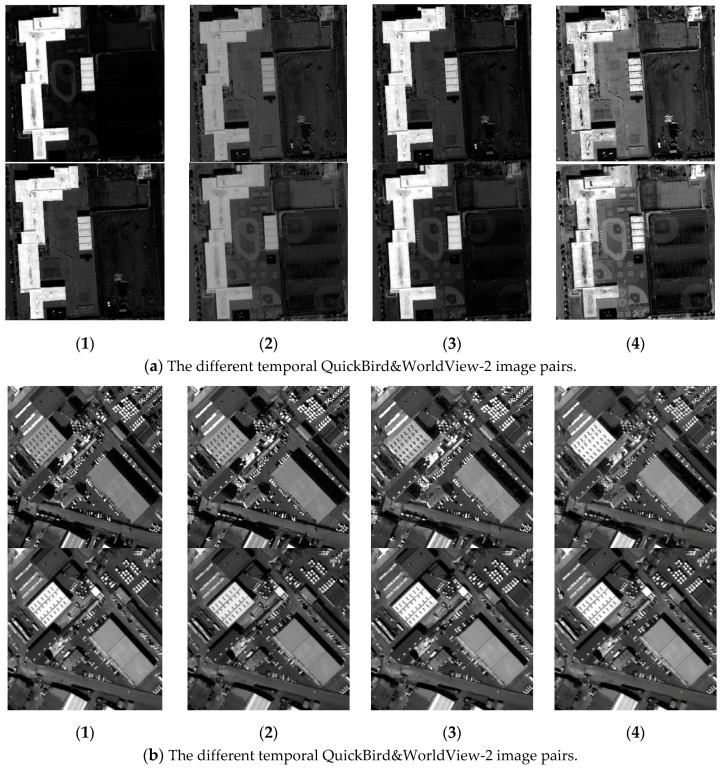

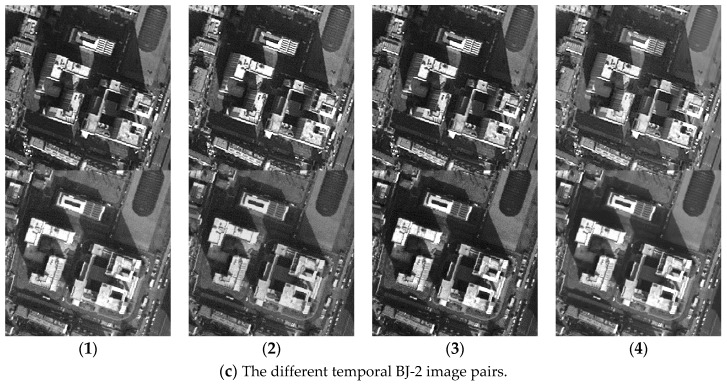
The comparative experiment results of radiation consistency on the mixed stereoscopic image pairs. (**a**) The different temporal QuickBird&WorldView-2 image pairs. (**b**) The different temporal QuickBird&WorldView-2 image pairs. (**c**) The different temporal BJ-2 image pairs. (Note: (**1**) The original images, (**2**) images by Zhang’s method, (**3**) images by Zhong’s method, (**4**) images by our method.)

**Figure 4:**
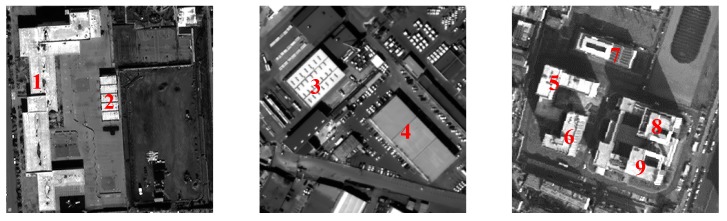
Object number.

Figure 3 shows comparative experimental results of radiation consistency for three groups of mixed stereoscopic image pairs consisting of different temporal and different sensors images. (1) in Figure 3 shows the original mixed stereoscopic image pairs, and (2), (3) in Figure 3 show the results by Zhang’s method [3] and by Zhong’s method [10], respectively. The results of our hierarchy radiation consistency based on objects are shown as (4) in Figure 3. Figure 4 shows corresponding object number mentioned in the experiments. Overall, the experimental results by three methods all obtain better radiation consistency than the original mixed stereoscopic image pairs. In order to prove the superiority of the proposed method for radiation consistency, we compare the experimental results by our method with the other two methods. The two compared methods both belong to radiometric normalization method. Zhang’s method proposed a two-layer illumination normalization method for contrast and brightness, respectively. Zhang’s contribution is multilevel processing for different illumination. Zhong’s method proposed hierarchical regression to build the linear relationship for suppressing the negative effect of change pixels. The main contribution is to build more accurate linear relationship for different temporal images. However, there are three obvious shortcomings in the radiation consistency results of the two other methods. (1) The compared two methods only focused on the radiometric normalization between the two images, and ignore the improvement of the quality for every single image. As shown in Figure 3a, Object 1 obviously has a phenomenon of overexposure, which leads to the reduction of the texture details itself and poor contrast in other objects. Our method includes the processing of illumination uniformity to improve the details of Object 1. (2) The two other methods have no processing for noise. From the Figure 3a,b, the similar smooth level is obtained by our method for the mixed stereoscopic image pairs of different sensors. Additionally, from Figure 3c, the quality is improved for the images of different times after our method. (3) The application aims of the two methods are change detection and mosaicking. The two compared methods mainly obtain grayscale consistency for the entire image. The aim of our method takes into account the grayscale consistency of every object for dense matching in detail. As shown in Figure 3a,b, Objects 2 and 3 obviously obtain the more similar radiometric properties using our method than the other two methods, which will be very helpful for dense matching. The proposed method in the paper comprehensively considered multiple radiation causes, which include illumination uniformity for every single image, illumination normalization, and similar smooth level between stereoscopic image pairs. It leads to our method having obvious advantages for radiation consistency recovery, especially in object areas.

### 6.3. The Analysis of Radiation Consistency Based on Objects

From the subjective analysis above, the two compared methods can adjust the overall illumination characteristics of stereoscopic image pairs to almost the similar level, while our method can recover not only the entire images but also object areas to similar level for illumination consistency and smoothness consistency. For the highly accurate 3D reconstruction, the grayscale consistency of object areas is important for dense matching. This group of experiments used improved histogram similarity for quantitative evaluation of the object radiation consistency. The formula of improved histogram similarity is shown as (20).

(20) $$C=1-\frac{[\max(j)-\min(j)]\cdot \sum_{j=0}^{255}\left|O_{1}(j)-O_{2}(j)\right|\xi_{j}}{2\cdot 255\cdot M\cdot \xi_{\max}}$$

in which *j* is the value of grayscale, $O_{1}(j)$ and $O_{2}(j)$ denote the number of pixels whose grayscale value are *j*, ξ(j)=|A−j|+1, A is the average value, $\xi_{\max}=\max(\left|A-j\right|+1)$, and *M* is the size of the image. When the *C* value is closer to 1, the two object images own the higher the consistency of the gray value distribution.

In order to reduce the influence of background information around the object areas, the minimum external rectangle of every object is cut to be used. Additionally, the background regions are set to 0. Table 1 shows the quantitative analysis of object radiation consistency, which is almost identical to the subjective evaluation above. From the table, the grayscale consistency of object 1 has a larger increase by our method than that by the two other algorithms. The main reason is that object 1 caused the phenomenon of overexposure in the original stereo image pairs, which leads to all pixels maintaining high gray level values and being in a small grayscale range interval. The results of the two methods are still in a very narrow gray range, which constitutes lack of texture details and poor uniformity. The improved histogram similarity increases penalties for image illumination uniformity, leading to the obtaining of better objective evaluation using our method. Objects 2 and 3 have a large difference of grayscale in the original image pairs. The compared methods mainly focus on the whole image, while our method proposed an object-oriented idea. As a result, the two objects obviously obtained higher radiation, consistent, experimental results using our method. Another point worth noting is that in Objects 5, 6, and 7, the similarity radiation of some results by the two methods are even lower than the original image pairs. This is due to the fact that global grayscale processing does not necessarily achieve better radiation consistency in the object areas. It is proved that the proposed algorithm in this paper has a great advantage and robustness in the recovery of radiation consistency in the object areas, which is very helpful for dense matching of objects in the urban areas. 

### 6.4. Comparative Experiments of Dense Matching for the Mixed Stereoscopic Image Pairs

**Figure 5:**
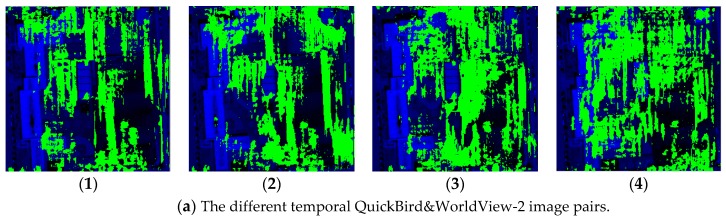

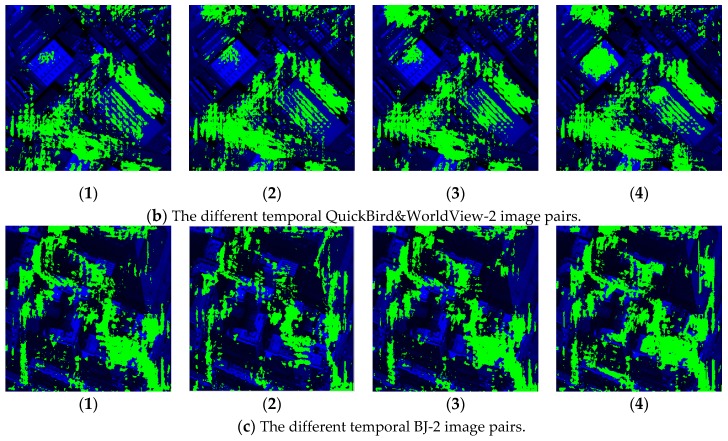
Comparative experiments of dense matching for the mixed stereoscopic image pairs. (**a**) The different temporal QuickBird&WorldView-2 image pairs. (**b**) The different temporal QuickBird&WorldView-2 image pairs. (**c**) The different temporal BJ-2 image pairs. (Note: (**1**) Dense matching results by the original images; (**2**), (**3**), and (**4**) correspond to dense matching results by Zhang’s method, Zhong’s method, and by our method, respectively.)

**Figure 6:**
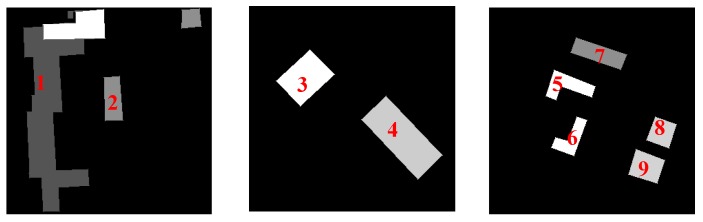
The truth image of disparity in object areas by manual.

Figure 5 shows dense matching results of the mixed stereoscopic image pairs, which are recovered by three radiation consistency methods. In Figure 5, green areas represent successful matching pixels, and blue areas are failed matching pixels and no matching pixels. The ground truth images of disparity map are shown as Figure 6. In the paper, when the difference between the matching results and the ground truth of disparity map is less than 1 pixel, the corresponding pixels are correct matching pixels that are labeled green. When the error is more than 1 pixel or the pixel is failed matching, that is labeled blue. From Figure 5, they are the matching results by the original mixed stereoscopic image pairs, after Zhang’s method, after Zhong’s method, and after our method.

From the subjective analysis, the correct matching rates of dense matching experiments are all relatively low. On the one hand, radiation quality between the two stereo image pairs still exists differences. On the other hand, it is the main reason that all the stereoscopic image pairs are different temporal images. Except for larger building objects, the background, roads, and some traffic have substantial changes, which lead to the changes in image content. In fact, the different contents cannot be matched, and the overall matching rates are all very low. Firstly, comparing (1) with (2)–(4) in Figure 5, the matching results after radiation consistency method are almost all better than the original generalized stereo pairs. As for the overall matching rate improved, it fully shows that the different radiation characteristics of the images have great influence on area-based dense matching. Secondly, comparing our method with the two other methods, the matching rates are obviously increased after our method, especially in the object areas. In the previous analysis, Objects 2 and 3 obtain far higher grayscale consistency by our method than by the two other methods. From Figure 5, the corresponding results are also obtained in the dense matching experiments. The two compared methods are both global radiation correction methods and ignore the specificity of the objects. Correspondingly, hardly any pixels are matched successfully in Objects 2 and 3. However, the proposed method in the paper greatly improved the radiation consistency of objects to obtain a high correct matching rate for object areas. In conclusion, the experimental results show that our hierarchy radiation consistency method is proposed to improve the dense matching ability of the mixed stereo image pairs, especially in the object areas.

In order to further demonstrate the advantages of the proposed object-oriented hierarchy radiation consistency algorithm on the dense matching of object areas, the quantitative analysis of correct matching rate in each object area is presented. As shown in Figure 6, the truth image of disparity in object areas is manually extracted. Additionally, the quantitative analysis of objects is shown in Table 2. From Table 2, the quantitative analysis results are similar to the analysis of radiation consistency based on objects. The correct matching rate of objects after radiation processing is almost increased. The correct matching rate of Objects 1 and 2 by our method is more greatly improved than the two compared methods. This is because Object 1 is overexposed to reduce texture details of both ObjectS 1 and 2, which leads to the low correct matching rate. The advantages of our hierarchy radiation consistency method include illumination uniformity to improve the radiation quality of Objects 1 and 2 in detail. The correct matching rate of Object 3 is not much improved by the two compared methods. The radiation consistency is failed to be recovered on Object 3 by the two methods, because they mainly focus on the global radiation consistency for change detection and image mosaicking. Our method is an object-oriented idea to recover more details for dense matching. The lower correct matching rate of Objects 5 and 6 is obtained by all the three methods. This is because the texture details of the original objects are relatively low, although the radiation consistency recovery of the two objects is great. There is no obvious texture feature on the top surface of objects that results in serious matching errors.

## 7. Conclusions

In this paper, we have put forward a novel hierarchy radiation consistency method for the mixed stereoscopic image pairs of different time and different sensor. Our work more fully considered multiple influence factors of different temporal and different sensor data on dense matching, which can control both the illumination consistency and the smoothness consistency. From the experiment results, our method obtained better consistency than the other two methods, especially in the object areas. Moreover, the dense matching experiment results proved once again that our method has a great advantage at acquiring the better correct matching rate of the mixed stereoscopic image pairs in object areas. The main contributions in the paper are summarized as follows:

1. A novel hierarchy radiation consistency method is proposed based on the comprehensive analysis of different temporal and different sensor data. For the different temporal stereoscopic image pairs, the illumination uniformity for single image and relative illumination normalization for two images are both considered to obtain the illumination consistency images. For the different sensor stereoscopic image pairs, different smoothness levels are solved by the proposed union group sparse method. Our hierarchy method simultaneously controls the illumination consistency and the smoothness consistency, which can be very helpful for dense matching.
2. The object-oriented method idea is proposed. The object extraction method and feature-based sparse matching method are employed to build a relationship between the same object areas in the mixed stereoscopic image pairs. Additionally, our radiation method can be carried out in the corresponding object areas, which can obtain the radiation consistency images in more detail. The object-oriented method idea is beneficial for the dense matching of building objects in urban areas.
3. In the smoothness consistency step, a union group sparse method is proposed based on the original group sparse model. The two different sensor images are improved to similar smoothness levels by the same threshold of singular value.

In conclusion, we proposed the object-oriented hierarchy radiation consistency method first, specifically for dense matching in the 3D reconstruction. The illumination consistency and the smoothness consistency images are obtained using our method, which can improve the application potential of different temporal and different sensor data in the 3D reconstruction field.

## Figures and Tables

**Table 1:** The quantitative analysis of radiation consistency based on objects.

	The Original Images	Zhang’s Method	Zhong’s Method	Our Method
Object 1	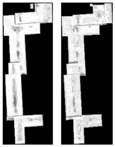	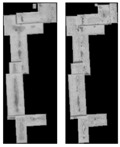	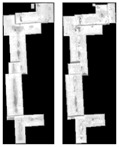	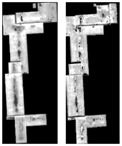
Similarity	0.748	0.750	0.763	**0.905**
Object 2	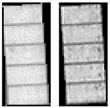	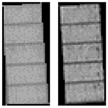	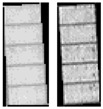	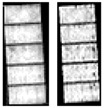
Similarity	0.915	0.944	0.930	**0.972**
Object 3	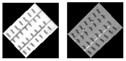	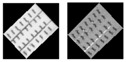	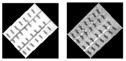	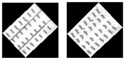
Similarity	0.468	0.656	0.643	**0.935**
Object 4	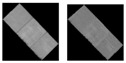	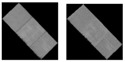	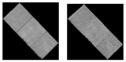	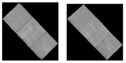
Similarity	0.907	0.973	0.966	**0.980**
Object 5	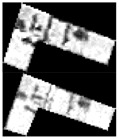	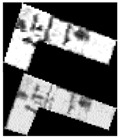	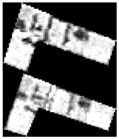	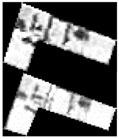
Similarity	0.888	0.777	0.855	**0.898**
Object 6	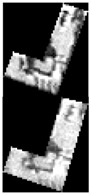	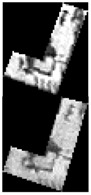	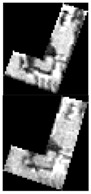	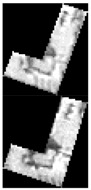
Similarity	0.851	0.765	0.856	**0.929**
Object 7	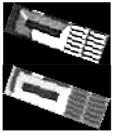	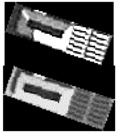	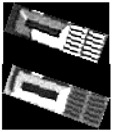	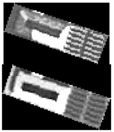
Similarity	0.814	0.769	0.835	**0.847**
Object 8	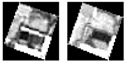	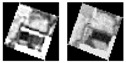	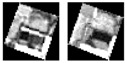	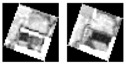
Similarity	0.837	0.857	0.811	**0.910**
Object 9	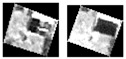	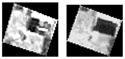	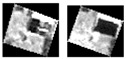	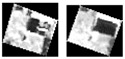
Similarity	0.838	0.804	0.859	**0.905**

**Table 2:** The correct matching rate in object areas.

Object Number	Origial Images	Zhang’s Method	Zhong’s Method	Our Method
1	0.074	0.111	0.062	0.449
2	0.000	0.064	0.063	0.737
3	0.060	0.176	0.240	0.821
4	0.597	0.577	0.602	0.619
5	0.271	0.033	0.293	0.376
6	0.124	0.013	0.131	0.327
7	0.345	0.126	0.345	0.505
8	0.602	0.607	0.612	0.613
9	0.510	0.473	0.682	0.699
